# Predictive analysis of lower limb fractures in the orthopedic complex operative unit using artificial intelligence: the case study of AOU Ruggi

**DOI:** 10.1038/s41598-022-26667-0

**Published:** 2022-12-22

**Authors:** Arianna Scala, Anna Borrelli, Giovanni Improta

**Affiliations:** 1 Department of Public Health, University of Naples “Federico II”, Naples, Italy; 2 San Giovanni di Dio e Ruggi d’Aragona” University Hospital, Salerno, Italy; 3 Interdepartmental Center for Research in Healthcare Management and Innovation in Healthcare (CIRMIS), Naples, Italy

**Keywords:** Health care, Engineering, Mathematics and computing

## Abstract

The length of stay (LOS) in hospital is one of the main parameters for evaluating the management of a health facility, of its departments in relation to the different specializations. Healthcare costs are in fact closely linked to this parameter as well as the profit margin. In the orthopedic field, the provision of this parameter is increasingly complex and of fundamental importance in order to be able to evaluate the planning of resources, the waiting times for any scheduled interventions and the management of the department and related surgical interventions. The purpose of this work is to predict and evaluate the LOS value using machine learning methods and applying multiple linear regression, starting from clinical data of patients hospitalized with lower limb fractures. The data were collected at the "San Giovanni di Dio e Ruggi d’Aragona" hospital in Salerno (Italy).

## Introduction

In the years from 2002 to 2019, the expenditure of the national health system underwent a great increase, going from 80 to 117 billion euros. Due to the various increases due to assistance, the need arose to evaluate methodologies for evaluating the reduction of assistance costs^1^.

One of the key strategies for jointly diminishing costs and hospital resource utilization concerns the Length Of Stay (LOS) management^2,3^. Furthermore, the LOS parameter is taken into consideration as a post-operative evaluation parameter in the assessment of quality in relation to health and care activities^4–7^. One of the areas of particular attention for the evaluation of LOS is the sector related to orthopedic activities. In fact, this work analyzes the hospitalizations relating to patients with fractures of the lower limbs. In the literature it has been reported that patients with orthopedic trauma undergo a majority of the hospital stay of about 10 days and therefore it is difficult to discriminate the trauma conditions that determine a greater hospital stay. Despite over time different protocols have been used and validated to allow post-operative care protocols to reduce LOS values, the differences associated complications such as infections make standardization of LOS difficult^8–10^.

Comorbidity, and therefore the presence of concurrent pathologies such as hypertension, anemia, fluid and electrolyte disturbances, is one of the main causes of an increase in the average LOS values, especially in the orthopedic sector. Only 4.9% of patients have no additional pathologies compared to the orthopedic trauma for which they are in the health facility^11^. It has been found that for patients with heart disease the LOS value increases compared to the mean LOS value^12^.

The hospital stay and therefore the value of the LOS and the related care costs are lower for patients who are admitted to the Day Hospital^13^. Therefore, in order to reduce hospital costs, it is necessary to plan the surgery as soon as possible which, according to what is reported by the studies in the literature, is also useful for improving the effectiveness of early intervention on the reduction of orthopedic surgery and other surgical processes^14–22^ In fact, according to the Italian surgical guidelines it is preferable to reduce the risk of complications and the relative stay in the health facility and the relative LOS value^23–26^.

Mathematical modeling was employed in the healthcare sector for several purposes: to optimize medical waste management processes in an Ethiopian hospital^27^; to forecast the propagation of viruses and bacteria^28,29^; to predict the LOS of patients undergoing valvuloplasty surgery^30^.

In the field of orthopedic surgery, machine learning algorithms and predictive models have been successfully applied which have proved to be optimal for the improvement of different health processes. Artificial neural network models, then compared with logistic regression models, were used to predict one-year mortality in elderly patients with intertrochanteric femoral fractures^31^. Machine learning (ML) was used for the analysis of healing times of lower limb fractures of children aged 0–12 years, using Random Forest and Self Organizing Feature Maps methods^32^. Neural networks and Random Forest were useful in selecting features for the evaluation of locomotor system degradation^33^. A multivariate logistic regression model was used to determine whether distal fractures of both upper and lower limbs occur in higher percentage in diabetic patients taking thiazolidinedione than in those not consuming it^34^.

The support of multi-criteria decision-making approaches^35–42^, along with more recent big data analyzes and simulations^43–53^, as well as advances in medical image and signal processing^54–58^, have enhanced the understanding of processes that affect healthcare costs and quality in order to design and integrate innovative methodologies to jointly diminishing cost, resource utilization and services quality^59–61^.

**Figure 1:**
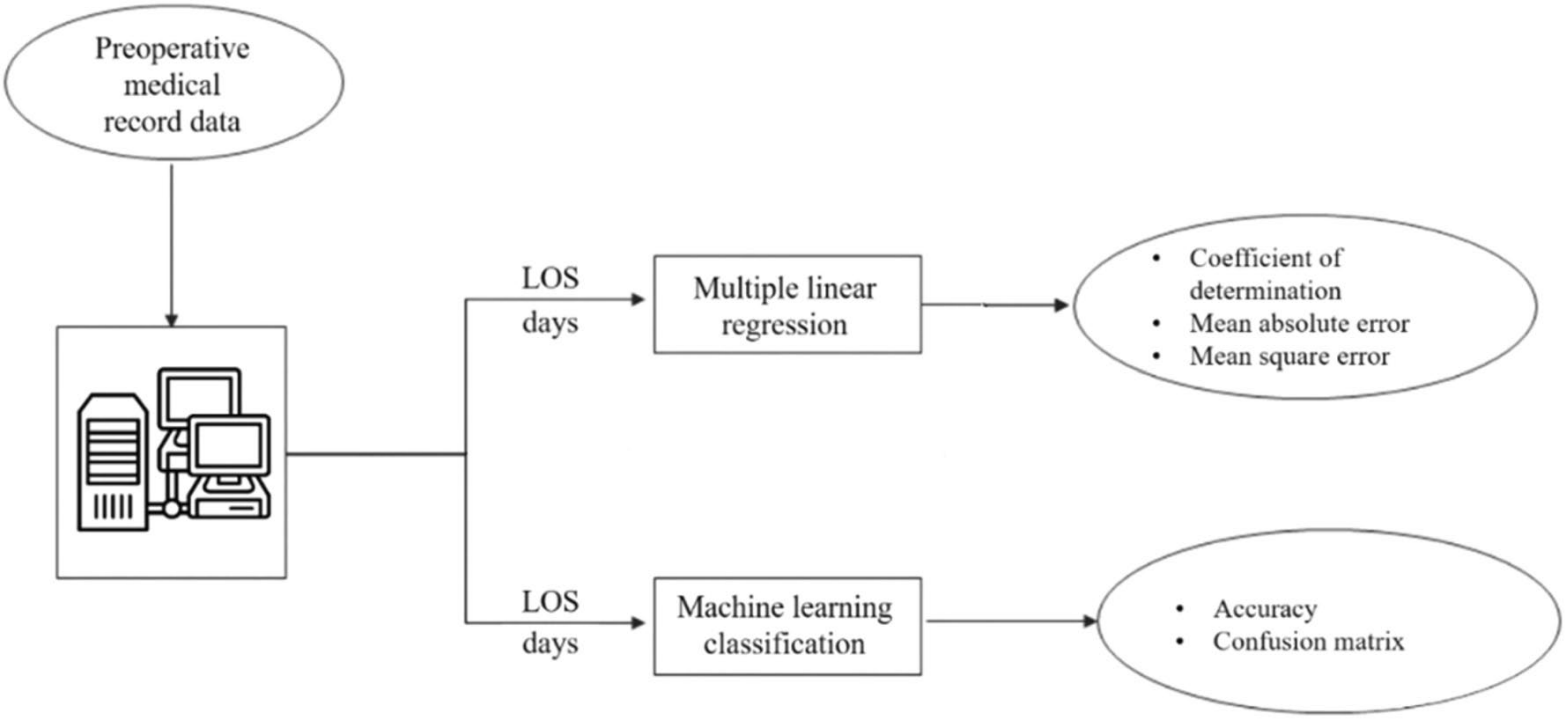
Study's workflow.

Our aim concerns the analysis of variables influencing the LOS of orthopedic patients; through the analysis of the medical records of the “San Giovanni di Dio e Ruggi d'Aragona” University Hospital of Salerno with particular attention to patients who in the years 2019 and 2020 were treated for having suffered fractures of the tibia and lower limbs. The collected data were used to model and predict overall hospital length of stay by following a two-way approach (Fig. 1): a multiple linear regression analysis and an ML classification analysis, performed to predict LOS clustered in weeks. Therefore, we designed different ML models (Random Forest, Decision Tree, Gradient Boosted Trees, Logistic Regression, Naïve Bayes and Support Vector Machine) trained on these data for making decisions. Our aim is to compute the prediction, of the LOS. Then, we discuss the potential of the model obtained as a tool for using hospital management. The present research work is both an extension and an improvement of a previous paper that the same authors presented at a conference^62^. An extension because the dataset considered is much larger both in terms of number of records and variables considered. An improvement because we have moved from classifying the length of hospital stay (LOS) in weeks to predicting it in a precise manner using regression techniques.

## Materials and methods

The dataset has been built by extracting information about 123 patients operated and hospitalized among 2019–2020 from the QuaniSDO informative system in use of the “San Giovanni di Dio e Ruggi d’Aragona”. The information collected for patients undergoing CS in the two hospitals considered is biographic (i.e., Gender or age), hospital (i.e., discharge or admission date) and clinical (i.e., comorbidities and complications during surgery) ones.

The different machine learning algorithms has been implemented by using Knime Analytics Platform to deal with the LOS task.

Furthermore, the dataset was expanded in order to extend the elaboration considering more patients and a major number of elements. The extracted date have been extracted by the "QuaniSDO" information system with the following inclusion criteria: "All patients with a principal diagnosis of lower limb fracture from 2011 to 2020 in both of the aforementioned departments".

In this manner, 706 hospital discharge forms were extracted with the following information for each patient:

- Year of discharge (2011–2020);
- Gender (Male/Female);
- Age;
- Department;
- Main diagnosis;
- Comorbidities;
- Diagnostic Related Group (DRG);
- Type of hospitalization;
- Type of procedure;
- Date of admission, discharge and procedure from which LOS and preoperative-LOS were obtained.

In order to create the multiple linear regression (MLR) model capable of predicting LOS, the following information was considered:

- Gender (Male/Female);
- Age;
- Department, encoded according to hospital rules with "3612" for Orthotraumatology and "3641" for Orthopaedics and Traumatology;
- Main diagnosis, encoded according to the ICD-9-CM (International Classification of Diseases-9th revision-Clinical Modification). All fractures of the lower limb were considered in the model:
  - 823.00: Closed fracture of upper end of tibia alone;
  - 823.02: Closed fracture of upper end of fibula with tibia;
  - 823.10: Open fracture of upper end of tibia alone;
  - 823.12: Open fracture of upper end of fibula with tibia;
  - 823.20: Unspecified fracture of shaft of unspecified tibia, initial encounter for closed fracture;
  - 823.22: Closed fracture of shaft of fibula with tibia;
  - 823.30: Open fracture of shaft of tibia alone;
  - 823.32: Open fracture of shaft of fibula with tibia;
  - 823.40: Torus fracture, tibia alone;
  - 823.42: Torus fracture, fibula with tibia;
  - 823.80: Unspecified fracture of shaft of right tibia, initial encounter for closed fracture;
  - 823.82: Closed fracture of unspecified part of fibula with tibia;
  - 823.92: Open fracture of unspecified part of fibula with tibia.
- Comorbidities (yes/no). Cardiovascular disease, hypertension, diabetes and obesity were considered.
- Type of hospitalization, encoded in hospital discharge forms as follows:
  - “1”: Planned admission, non-urgent;
  - “2”: Urgent admission.
- Type of procedure, encoded according to the ICD-9-CM:
  - 77.07: Sequestrectomy, tibia and fibula;
  - 77.49: Biopsy of bone; other;
  - 78.10: Application of external fixator device; unspecified site;
  - 78.14: Application of external fixator device, carpals and metacarpals;
  - 78.15: Application of external fixator device, femur;
  - 78.17: Other Operations On Bones, Except Facial Bones;
  - 78.59: Internal fixation of bone without fracture reduction, other bones;
  - 78.67: Removal of implanted devices from bone, tibia and fibula:
  - 79.00: Closed reduction of fracture without internal fixation, unspecified site;
  - 79.06: Closed reduction of fracture without internal fixation, tibia and fibula;
  - 79.16: Closed reduction of fracture with internal fixation, tibia and fibula;
  - 79.30: Open reduction of fracture with internal fixation, unspecified site;
  - 79.36: Open reduction of fracture with internal fixation; tibia and fibula;
  - 93.53: Application of other cast;
  - 93.54: Application of splint.
- Preoperative-LOS.

### Machine learning algorithms

The ML models can be divided into supervised, that learns from historical data to classify the sample in the inference phase, and unsupervised, that aims to find some hidden pattern to cluster all the samples. In this section, we discuss about different machine learning models used for our analysis, that fall in the first category, whose learning phase (also called training phase) has been made on a set of entire samples (usually 80%) whilst the remain part is used for evaluating the designed model (test/inference phase). We used the Decision (DT) being an algorithm that bases prediction on the construction of decision trees. In each node, a condition is verified and, according to the value assumed by one of the features, a path is determined through one of the branches. This is done until a value is assigned to the target variable. The Random Forest (RF) and Gradient Boosted Trees (GBT) rely on the tree data structure, but use a set of it in order to improve the performance of the single, used by DT. In this way, it is possible to build a strong predictive model, although overfitting problems can be generated. RF and GBT. Naïve Bayes (NB) learns from historical information by using the Naïve Bayes theorem. Finally, Support Vector Machine (SVM) is based on the construction of a hyperplane that separates the different classes identified in the training phase. Therefore, it has a more complex structure of DT. It is widely used on non-linear and small data sets. In addition, the 3 algorithms that had the highest accuracy in the conference paper were used ed in particular 75% Training and 25% testing.

In this case, LOS was divided into weeks:

- Group 1: from 1 to 7 days;
- Group 2: from 8 to 14 days;
- Group 3: > 14 days.

### Statistical analysis

The aim of MLR unveils hidden relationships between regressors, representing the input variables, and output variables in order to analyze the predictions. The equation representing regression can be written as follows:

$$ {\text{y}} = \upbeta_{0} + \upbeta_{1} {\text{x}}_{1} + \upbeta_{2} {\text{x}}_{2} + \upbeta_{3} {\text{x}}_{3} + \upbeta_{4} {\text{x}}_{4} + \upepsilon $$

in which we can note that:

- y corresponds the LOS value;
- β_0_ is intercept value;
- xi are the independent variables;
- β_i_ are the estimated regression coefficients of respective variables;
- ε is the regression error.

### Ethics aproval

In compliance with the Declaration of Helsinki and with the Italian Legislative Decree 211/2003, Implementation of the 2001/20/CE directive, since no patients/children were involved in the study, the signed informed consent form and the ethical approval are not mandatory for these type of studies. Furthermore, in compliance with the regulations of the Italian National Institute of Health, our study is not reported among those needing assessment by the Ethical Committee of the Italian National Institute of Health.

## Results

In the previous paper [Colella et al.^62^] the algorithms implemented were those in Table 1.

**Table 1:** Effectiveness results of ML models.

Models	Accuracy (%)	Error (%)
DT	84.00	16.00
RF	88.00	12.00
SVM	88.00	12.00
GBT	72.00	28.00
LR	88.00	12.00
NB	92.00	8.00

The best performance was obtained with NB algorithm with an accuracy equals to 92%.

Following the results obtained with the extended dataset are presented.

In particular, in Table 2 is shown a distribution of the different characteristics for the 706 accesses of the dataset considering different type of parameters.

**Table 2:** Distribution of the features into the sample of the extended Dataset of 706 accesses.

Features	Dataset (N = 706)
**Gender**	
M	463
F	243
**Age**	
Age ≤ 45	328
45 < Age ≤ 65	260
Age > 65	118
**Department**	
3612	519
3641	187
**Main diagnosis**	
823.00	183
823.02	70
823.10	2
823.12	3
823.20	118
823.22	245
823.30	14
823.32	28
823.40	11
823.42	8
823.80	8
823.82	13
823.92	3
**Comorbidities**	
Yes	60
No	646
**Type of hospitalization**	
1	8
2	698
**Type of procedure**	
77.07	6
77.49	8
78.10	5
78.14	5
78.15	4
78.17	53
78.59	7
78.67	10
79.00	22
79.06	56
79.16	59
79.30	19
79.36	399
93.53	17
93.54	36

Table 3 shows the characteristics of the regression model.

**Table 3:** Evaluation metrics for the regression analysis of LOS measured in days.

Model summary^b^					
Model	R	R square	Adjusted R square	Std. error of the estimate	Durbin–Watson
1	0.897a	0.805	0.802	1.902	1.834

An Adjusted R square of 0.805 is a good value and shows that the model is able to predict LOS adequately. The standard error is 1.902 while the Durbin-Watson test between 1.5 and 2.5 indicates that the values of the residuals are independent, a fundamental assumption for the model to be developed.

A Fisher's test was performed to assess the joint significance of the coefficients. The p-value is less than 0.05 so the model has explanatory power (Table 4).

**Table 4:** Fisher test.

ANOVA^a^					
Model	Sum of squares	df	Mean square	F	p-value
**1**					
Regression	10,381.178	8	1297.647	358.829	0.000b
Residual	2520.589	697	3.616		
Total	12,901.768	705			

Table 5 shows, for each independent variable, the coefficients obtained, the t-test and the p-value obtained. The test is significant for p-value < 0.05 for which age, gender, department, type of hospitalization and pre-los significantly influence LOS.

**Table 5:** Regression coefficient and t-test.

Coefficients^a^					
Model	Unstandardized coefficients		Standardized coefficients	t	p-value
	B	Std. error	Beta		
**1**					
(Constant)	232.223	355.431		0.653	0.514
Age	0.017	0.004	0.079	4.340	0.000
Gender	− 0.325	0.156	− 0.036	− 2.086	0.037
Department	− 0.018	0.006	− 0.052	− 3.009	0.003
MainDiagnosis	− 0.002	0.004	− 0.008	− 0.472	0.637
Procedure	0.000	0.000	− 0.013	− 0.763	0.446
Comorbidities	− 0.183	0.276	− 0.012	− 0.663	0.507
Type of hospitalization	1.594	0.682	0.039	2.338	0.020
pre-operative LOS	0.923	0.018	0.875	51.082	0.000

As can be seen from Table 6, we can note that VIF < 10 and tolerance > 0.2 so it can be said that there is no multicollinearity in the data, another fundamental assumption for the model to be developed.

**Table 6:** Evaluation of collinearity statistics.

Coefficients^a^		
Model	Collinearity statistics	
	Tolerance	VIF
**1**		
(Constant)		
Age	0.835	1.197
Gender	0.934	1.071
Department	0.923	1.084
MainDiagnosis	0.981	1.019
Procedure	0.992	1.008
Comorbidities	0.866	1.155
TypeOfHospitalization	0.983	1.017
PreOperativeLOS	0.956	1.046

**Figure 2:**
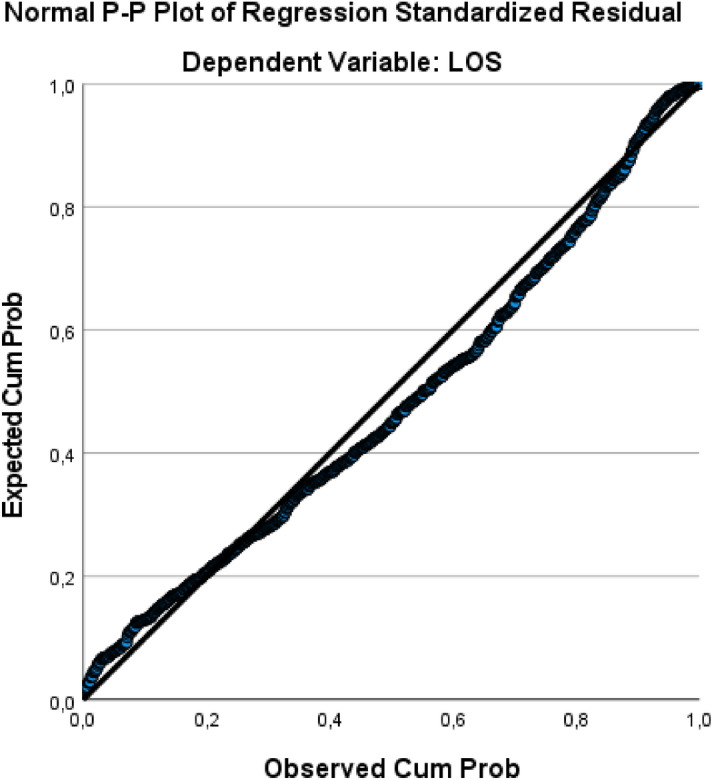
P-P plot Regression.

Figure 2 shows how the residual values are normally distributed as points are almost all on the diagonal.

**Figure 3:**
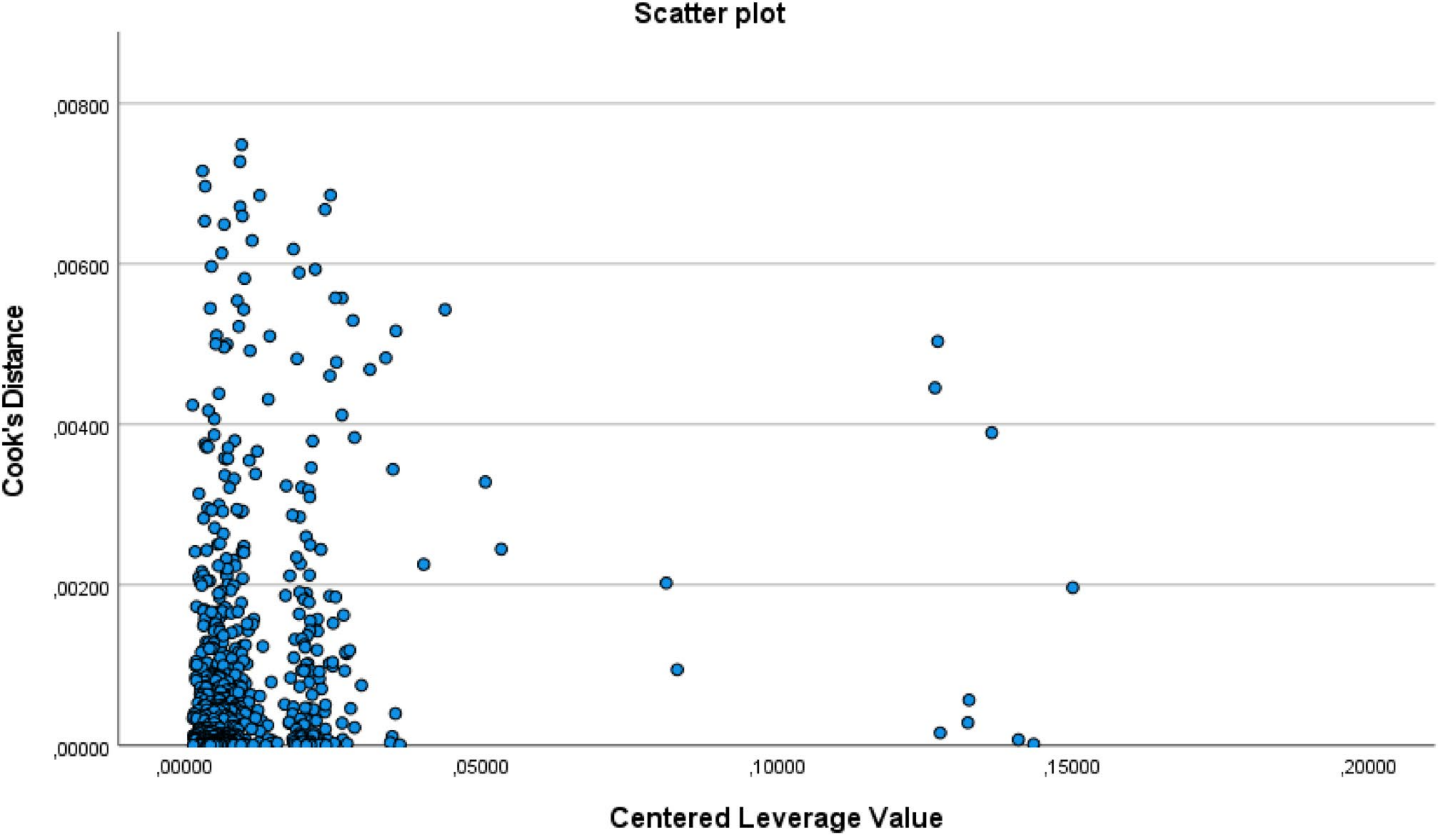
Scanner plot related for the evaluation of Cook’s Distance values.

As it is possible to see in the Fig. 3 all Cook distance values are much less than 1 so there are no outliers affecting the model.

In the end, having considered more variables and a longer time frame, the 3 ML algorithms were also used which in the conference paper were better to understand how the accuracy varied (Table 7). The results of ML analysis are presented in terms of accuracy, precision, sensitivity, specificity and F-measure.

**Table 7:** Performance metrics of ML algorithms.

Performance metrics	Class	RF	LR	NB
Accuracy (%)	Overall	75.7	70.6	75.1
Error (%)	Overall	24.3	29.4	24.9
Precision (%)	1	76.8	71.4	73.9
	2	73.8	68.8	74.7
	3	100	100	90
Sensitivity (%)	1	89	85.4	82.9
	2	72.8	65.4	69.1
	3	14.3	14.3	64.3
Specificity (%)	1	76.8	70.5	74.7
	2	78.1	75	80.2
	3	100	100	99.4
F-measure (%)	1	82.5	77.8	78.2
	2	73.3	67.1	71.8
	3	25	25	75

1 $$Accuracy=\frac{n^\circ \,\,of \,\,correct \,\,predictions}{total \,\,n^\circ \,\,of \,\,predictions}$$

2 $$Precision=\frac{n^\circ\,\, of \,\,true \,\,positives}{n^\circ \,\,of \,\,true \,\,positives + n^\circ \,\,of \,\,true \,\,negatives}$$

3 $$Sensitivity=\frac{n^\circ \,\, of\,\, true \,\,positives}{n^\circ \,\,of \,\,true \,\,positives + n^\circ \,\,of \,\,false \,\,negatives}$$

4 $$Specificity=\frac{n^\circ\,\, of \,\,true \,\,negatives}{n^\circ \,\,of \,\,true \,\,negatives + n^\circ \,\,of \,\,false \,\,positives}$$

5 $$F-measure=2*\frac{(precision*recall)}{precision +recall}$$

Table 8 shows the RF confusion matrix, in which we can note that 134 out of 177 predictions were correct, namely those on the diagonal of the matrix.

**Table 8:** RF model confusion matrix.

Real/predicted	1	2	3
1	73	9	0
2	22	59	0
3	0	12	2

**Figure 4:**
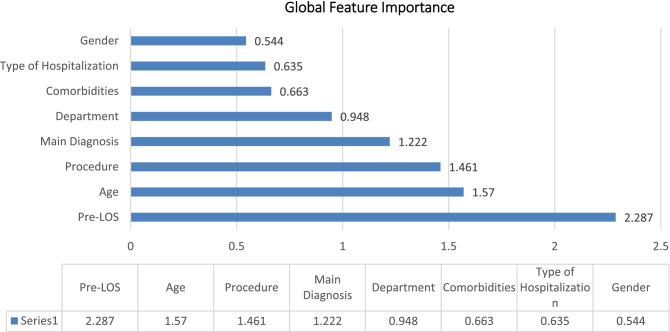
Global feature importance.

Finally, a global surrogate Random Forest was used, which is a Random Forest model trained to approximate the predictions of the original model. The Random Forest was trained on pre-processed input data in a standard way with the optimized parameters "Tree Depth," "Number of Patterns," and "Minimum Size of Child Nodes." The surrogate model was successfully trained with an accuracy of 0.986 with respect to the class of interest predicted by the original model "LOS week: 1." Feature importance is calculated by counting how many times it was selected for a split and at what level (level) among all available (candidate) features in the random forest trees. A higher value indicates greater feature importance (Fig. 4).

Preoperative-LOS was obviously the most significant feature, followed by age, procedure, and principal diagnosis.

## Discussion and conclusion

In this paper, our aim is to investigate the LOS prediction task, whose aim is to jointly diminish the hospital resource and costs in order to support the decision making process of managers; in fact, improving the LOS prediction allows to enhance bed estimation to focus the hospital resources mainly on the subjects affecting by several disease, also decreasing their occupancies. The LOS prediction task can be further useful for several applications (i.e., reimbursement or accounting^63,64^.

For this reason, the MLR and several ML models have been designed and appropriately trained to predict LOS of subjects under lower limb parameters. Our experimental evaluation made over a large cohort of patients shows that the RF achieves highest results in accuracy (75.7%) in predicting LOS. So taking into account a larger dataset with more accesses but also with more variables, the ML algorithms returned lower accuracy than the previous work which had an accuracy of 88%^62^. The MLR model with an R-square of 0.80 proves to be a valid decision support for this type of patient. This task further can support the hospital resources in their decision-making process. This type of "double analysis" has already been performed to predict LOS of patients who have undergone femur fracture [54.] In fact, the first analysis is performed with MLR that predicts LOS in a punctual way and the second analysis instead uses different ML algorithms classifying LOS in weeks (3 groups). As in the aforementioned study, the ML results are good with accuracy above 75%. As for MLR in our case the model is superior with a much higher R-square.

As it was possible to see from the results, the development of the elaboration created starting from the additional dataset takes into consideration a greater number of variables than the starting one as well as a greater number of accesses considered. The results show a significant influence of age, gender, department, type of hospitalization and pre-los for the increasing of LOS (Table 5).

A comparison of the significance of the regression coefficients (Table 5) and the importance ranking of the characteristics (Fig. 4) obtained by applying the machine learning models reveals that the most influential factors, according to the RF algorithm, are preoperative LOS, age, and procedure type, which only partially overlaps with the significance of the regression coefficients. In fact, preoperative LOS and age proved to be significant predictors in both multiple regression and machine learning models, while procedure type assumed greater significance as a predictor of LOS in ML analysis than in regression analysis. Finally, ward, type of hospitalization, and sex were significant for the regression analysis but not very significant for the ML algorithms. These results would recommend that the interpretation of predictive models of the healthcare process should be done with caution and in consideration of the value and effect of the predictors chosen and used in the models. In fact, comparing the relevance of the predictors in the regression and classification models examined is an essential part of assessing the validity of the results and should be the guide for obtaining reasonable and interpretable results when dealing with predictive algorithms in the healthcare context.

The additional variables takes into account the related parameters allows to enhance the performance of the proposed approach over a cohort of subjects under lower limb fractures although it can improve the complexity of the entire system.

## Data Availability

The datasets generated and/or analyzed during the current study are not publicly available for privacy reasons but could be made available from the corresponding author on reasonable request.
